# Emergy Analysis and Sustainability Efficiency Analysis of Different Crop-Based Biodiesel in Life Cycle Perspective

**DOI:** 10.1155/2013/918514

**Published:** 2013-05-14

**Authors:** Jingzheng Ren, Alessandro Manzardo, Anna Mazzi, Andrea Fedele, Antonio Scipioni

**Affiliations:** Department of Industrial Engineering, Quality and Environmental Research Centre (CESQA), University of Padova, Via Marzolo 9, 35131 Padova, Italy

## Abstract

Biodiesel as a promising alternative energy resource has been a hot spot in chemical engineering nowadays, but there is also an argument about the sustainability of biodiesel. In order to analyze the sustainability of biodiesel production systems and select the most sustainable scenario, various kinds of crop-based biodiesel including soybean-, rapeseed-, sunflower-, jatropha- and palm-based biodiesel production options are studied by emergy analysis; soybean-based scenario is recognized as the most sustainable scenario that should be chosen for further study in China. DEA method is used to evaluate the sustainability efficiencies of these options, and the biodiesel production systems based on soybean, sunflower, and palm are considered as DEA efficient, whereas rapeseed-based and jatropha-based scenarios are needed to be improved, and the improved methods have also been specified.

## 1. Introduction

With the depletion of resource, the shortage of energy, and the pollution of the environment, renewable and clean energy has gained more and more attentions nowadays. Being crop based, biodiesel that is a renewable fuel and has lower environmental impact has become a growing rapidly industry in the past decades [1]. However, this positive image has changed dramatically in the last few years, due to several reasons: (i) the diversion of large proportions of grains from food to biodiesel production has caused the increase in the food price; (ii) the global ecological performance of biodiesel is worse than that of fossil fuel; (iii) “the fuel versus food” debate [2, 3].

Biodiesel can be produced from oilseeds such as soybean, rapeseed, sunflower, palm oil, and jatropha seeds [4]. Different kinds of crop-based biodiesel have different performance on economic, environmental, and social aspects. Economy, environment, and society are the three pillars of sustainability [3]. Therefore, it can be concluded that different crop-based biodiesel have different sustainability. In order to fulfill the concept of sustainable development, selecting the most sustainable crop-based biodiesel is of vital importance.

Several methods or combinations of some of them have been applied to evaluate the sustainability of biofuel in recent years, such as life cycle assessment [11–14], multicriteria decision-making [15–17], ecological footprint [18], and emergy analysis [19–21]. Among these methods, emergy analysis is the most direct and apparent method to identify the sustainability of an industrial system, because the emergy index of sustainability in emergy analysis is a measure of the sustainability of the industrial systems.

Emergy is the available energy of one kind that has been used up directly and indirectly to make a product or service [22]. The use of a common basis (solar equivalent joules, (sej)) permits to account, all the energy contribution to obtain a certain product or service [23]. It is a powerful tool that can measure the real wealth of the work of nature and society and assess public policies aimed at sustainability and fair trade [21]. Although there are some published papers using emergy analysis as tool to measure the feasibility and sustainability of various crops for biodiesel production, these studies are only about the assessment of the sustainability of the crops or one crop-based biodiesel. Therefore, in order to evaluate the sustainability of various crops-based biodiesel comprehensively, using emergy analysis to evaluate the sustainability of various crops-based biodiesel in life cycle perspective is prerequisite.

Emergy analysis is used to study the rapeseed-, sunflower-, soybean-, jatropha-, and palm-based biodiesel, and DEA is used to evaluate the sustainability efficiency in this paper. This paper is organized as follows: Section 2 is emergy analysis; Section 3 is sustainability efficiency analysis, followed by Section 4. 

## 2. Emergy Analysis of Various Crops-Based Biodiesel

### 2.1. Emergy Analysis

Emergy analysis has superiority compared with energy analysis or economic analysis, and it cannot only reflect the quality of energy, but also calculate different kinds of energy together on the common unit of joule [5]. Emergy represents solar equivalent joules (sej) to measure natural and economic resources [6]. Solar emergy is usually measured in solar emergy joules (sej), and solar transformity is expressed as solar emergy joules per joule of product (sej/J). When an item is expressed in units different than joules, for instance grams, then the quality factor is energy/mass (sej/g). The formula for the calculation of emergy has been shown in (1).

**Figure 1:**
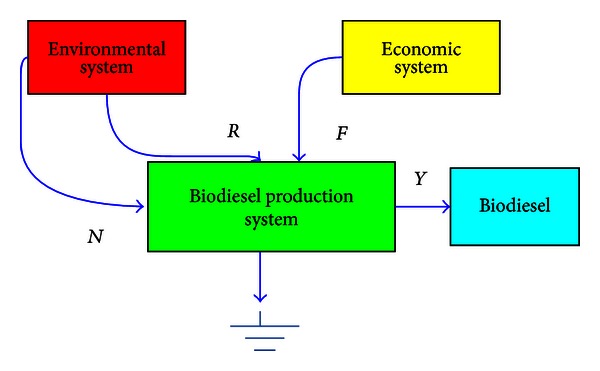
Inputs and outputs emergy flows of the biodiesel production system.

The inputs and outputs of emergy flows of a system are shown in Figure 1 and the emergy indices and corresponding calculated methodology used in this paper are shown in Table 1.

Total emergy assigned to the output is called emergy yield (*Y*), and it is the sum of renewable environmental resources (*R*), nonrenewable environmental resources (*N*) and purchased feedback flows of goods and human services from the economy (*F*). Several other indicators such as transformity (Tr), emergy yield ratio (EYR), emergy investment ratio (EIR), environmental loading ratio (ELR) and environmental sustainability index (ESI) have been used to evaluate the global performance of the systems.

The emergy yield ratio (EYR) provides insight into the net benefit of the various production processes to society, it can be used to measure the ability of the process to rely on local resources, and it does not make any difference local and imported (purchased or “invested”) emergy flows [24]. The environmental loading ratio (ELR) can provide additional information to EYR, it expresses the use of environmental services by a system and reflects the impact on environmental stress [25]. Environmental investment ratio (EIR) shows the relation between the emergy of the economic inputs with those provided by the environment, renewable or not [23]. Emergy index of sustainability (EIS) is an aggregate measure of yield and environmental loading, that is, a sustainability function for a given process (or economy) under study [24].

(1) Emergy=available energy of item×transformity.

### 2.2. Emergetic Ternary Diagram

**Figure 2:**
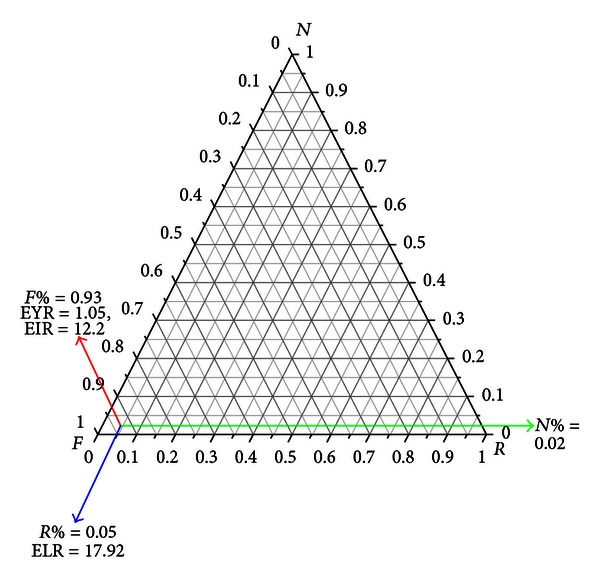
Resource flow lines in the emergetic ternary diagram.

Emergetic ternary diagram, including resource flow lines, sensitivity lines, and sustainability line, has been developed by Almeida et al. [26] (Figure 2). With the emergetic ternary diagram, the sustainability of different options can be compared graphically.

Resource flow lines are ternary combinations represented by points within the triangle, the relative proportions of the elements being given by the lengths of the perpendiculars from the given point to the side of the triangle opposite the appropriate element. A resource flow line with the proportions of  *R*,  *N*,  *F*  0.05, 0.93, and 0.02 respectively, is shown in Figure 4.

**Figure 3:**
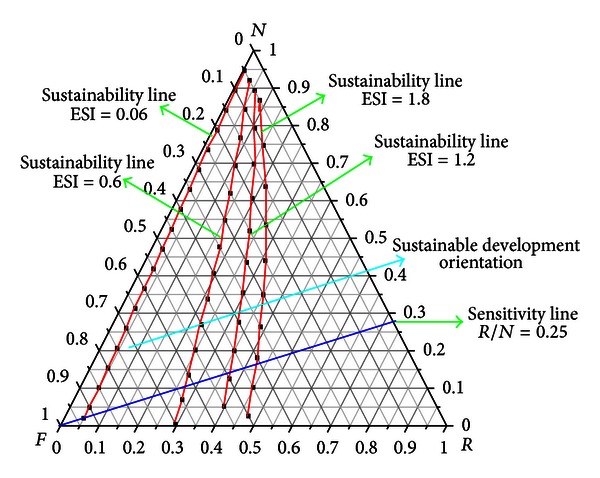
Sustainability line in emergetic ternary diagram.

Sustainability line is a line which indicates the sustainability index, and it departs from the *N* apex in the direction of the *RF* side allowing the division of the triangle into sustainability areas. The sustainability lines with the emergy index of sustainability 0.06, 0.6, 1.2, and 1.8, respectively, are shown in Figure 3. Each system on the same sustainability line has the same emergy index of sustainability, and the nearer the line to *R* apex, the bigger the emergy index of sustainability. The direction in which the system of the emergy index of sustainability will be bigger is called sustainable development orientation.

Sensitivity line is the line that follows the variation of a given resource flux (*R*,  *N*, or  *F*), and any points along the line represent a condition in which the other two fluxes remain in the same initial proportion [23]. A sensitive line in which the ratio of *R* and *N* has the value of 0.25 is shown in Figure 3:

(2) ESI=EYRELR=(R+N+F)/F(N+F)/R,R+N+F=100%,ESI=1−F−NF2+F×N.

On the basis of (2), *R* and *N* can be formulated by ESI and *F* in (3):

(3) R=ESI×F1+ESI×F,N=11+ESI×F−F.

*N* is always positive and should satisfy (4).

(4) 11+ESI×F−F≥0.

Therefore, ESI is fixed, *F* should satisfy (5), and the maximal proportion of economic inputs is a certain value:

(5) F≤1+4ESI−12ESI.

For instance, if ESI = 0.06, the maximal proportion of economic inputs cannot exceed 94.63% in order to satisfy the sustainable target.

When *F* is fixed, ESI should satisfy (6). It means that when the proportion of *F* equals 94.63%, the maximal Emergy sustainable index is 0.06:

(6) ESI≤1−FF2.

When ESI < 1, products and process are not sustainable in a long term, when 1 < ESI < 5 the products or processes may have a sustainable contribution to the economy for medium periods, and when ESI > 5 they can be recognized as sustainable in a long term, but if ESI > 10, the processes are underdeveloped [23].

Based on (5), it can be deduced that the proportion of purchased emergy is: (i) more than 61.80%, the process is not sustainable in a long term, (ii) between 35.83% and 61.80%, the processes may have a sustainable contribution to the economy for medium periods, (iii) lower than 35.83%, it is sustainable in a long term, nevertheless the higher ESI value does not meant the process with better sustainability, and (iv) below 27.02%, the whole system is underdeveloped.

### 2.3. Function Unit and Boundary of the System

**Figure 4:**
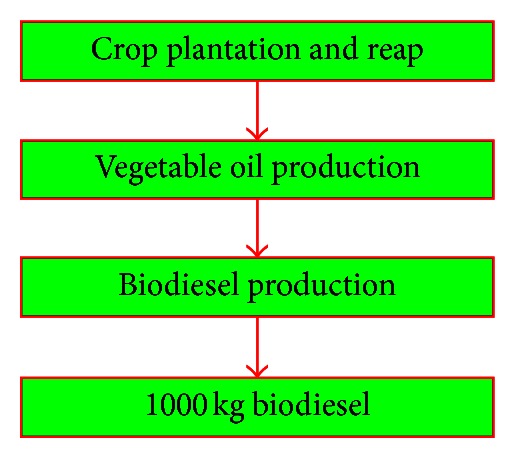
The system boundary of the biodiesel production system based on life cycle prespective.

The hypothetical systems for biodiesel production are located in China, and the statistical data are based on the average level of China. The functional unit in this study is 1000 kg biodiesel and the system boundary is shown in Figure 4; the latter consists of crop plantation and reap, vegetable oil production, and biodiesel production.

### 2.4. Emergy Analysis of Biodiesel

Five kinds of crop-based biodiesel options including soybean-, rapeseed-, sunflower-, palm-and jatropha-based options were studied with emergy analysis, and the alkali-catalyzed process is used to produce biodiesel in the current conditions of China. The emergy of each item can be calculated by (1). The available energy of each item has been calculated in three ways: (i) calculation with the published works, (ii) estimation with the known information, and (iii) statistics. Some of the data in the references have been adjusted according to the suggestions of technical staff of farm science station and engineers of crop oil factories and biodiesel factories.

The emergy analysis of 1000 kg soybean-based biodiesel has been taken as an example to show how to obtain the available energy of each item. With the survey and the data provided by Yang et al. [5], Tsoutsos et al. [27], and Zhang et al. [28], it can be deduced that 1 kg soybean oil needs 5.88 kg soybean, 1 kg soybean-based biodiesel needs 1.0068 kg soybean oil, and the yield of soybean is 2540 kg/ha; therefore, 23304.59 m^2^ of crop land is needed for producing 1000 kg soybean-based biodiesel.

**Figure 5:**
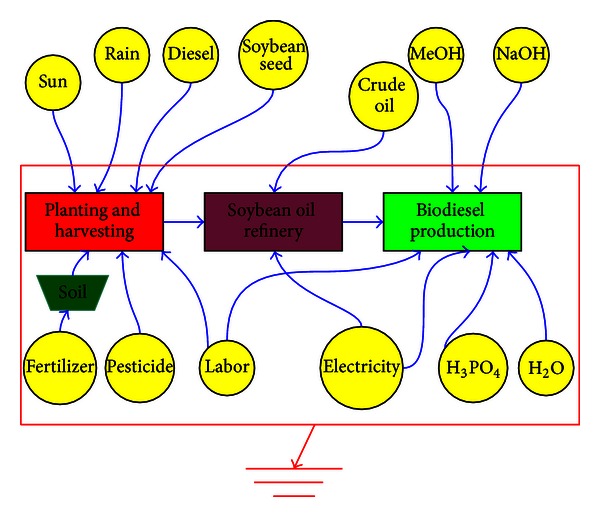
Emergy flow diagram of soybean-based biodiesel production system.

The emergy flow diagram of soybean-based biodiesel production system has been shown in Figure 5. Then, the available energy of sunlight, rain, wind, and topsoil loss land can be calculated as shown in Table 2, then with the transformity of each item and (1), the emergy of each item in soybean-based biodiesel system can be obtained, as shown in Table 3.

Similarly, with the survey and the data provided by [27–33], the emergy analysis tables of rapeseed-based, sunflower-based, palm-based, and jatropha-based biodiesel have been shown in Tables 4, 5, 6, and 7, respectively. The emergy indices of various crop-based biodiesel have been shown in Table 8.

The emergy indices of sustainability of all the crop-based biodiesel studied in this paper are lower than 1 and approach 0; it means that these scenarios for biodiesel production are not sustainable in a long term.

The transformities of soybean-based, rapeseed-based, sunflower-based, palm-based, and jatropha-based biodiesel productions are calculated to be 9.85*E *  + 12 sej/kg, 9.18*E *  + 12 sej/kg, 6.40*E *  + 12 sej/kg, 5.83*E *  + 12 sej/kg, 1.61*E *  + 13 sej/kg, respectively and palm-based biodiesel is the most emergy-saving option, and jatropha is the least.

The sequence of the emergy yield ratios from the biggest to the smallest is soybean, sunflower, rapeseed, palm, and jatropha, to some extent, it indicates that this index can represent the proportion of the economic inputs, the smaller the index, the more the system depends on purchased inputs.

The sequence of the environment load ratio from the biggest to the smallest is jatropha, palm, rapeseed, sunflower, and soybean, and it means that soybean-based biodiesel has the best environmental performance. On the contrary, jatropha-based biodiesel has the worst environmental performance. And the sequence of environmental investment ratio is the same as sequence of the environment load ratio, and it denotes that the economic inputs in jatropha-based biodiesel system occupies a higher proportion in the total solar emjoules than the other four, jatropha-based biodiesel system dependents on the purchased inputs significantly.

**Figure 6:**
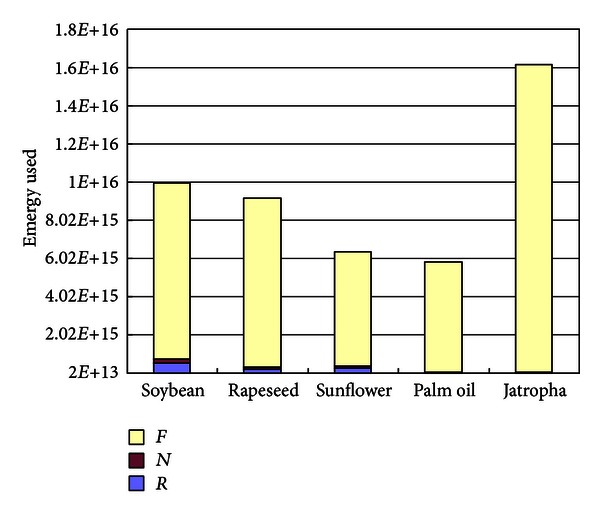
The solar emjoules of each crop-based biodiesel system.

The solar emjoules of each crop-based biodiesel system is shown in Figure 6. It is apparent that economic inputs occupy a very high proportion in each system, and palm-based biodiesel consumes the least solar energy when taking palm seeds as feedstock to produce biodiesel. It could also be concluded that the total consumed emergy for various crop-based biodiesel depend on the purchased inputs significantly.

**Figure 7:**
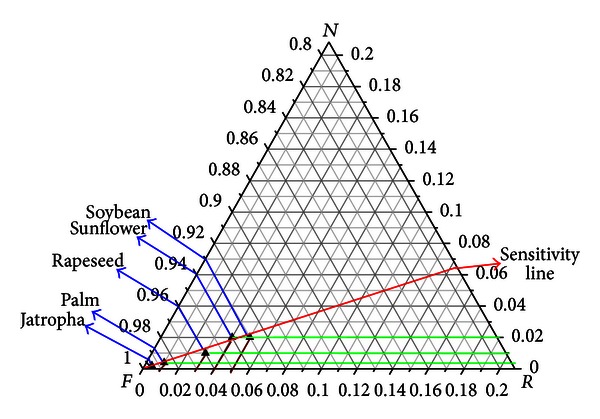
Resource flow lines of various crops-based biodiesel in the emergetic ternary diagram.

The resource flow lines of various crops based biodiesel in the emergetic ternary diagram are shown in Figure 7. The sustainability sequence of the five kinds of biodiesel production systems from the best to the worst is palm, rapeseed, sunflower, soybean and jatropha. The biodiesel based on palm-based system has been recognized as the most sustainable, and the sustainability of jatropha-based system is the worst.

**Figure 8:**
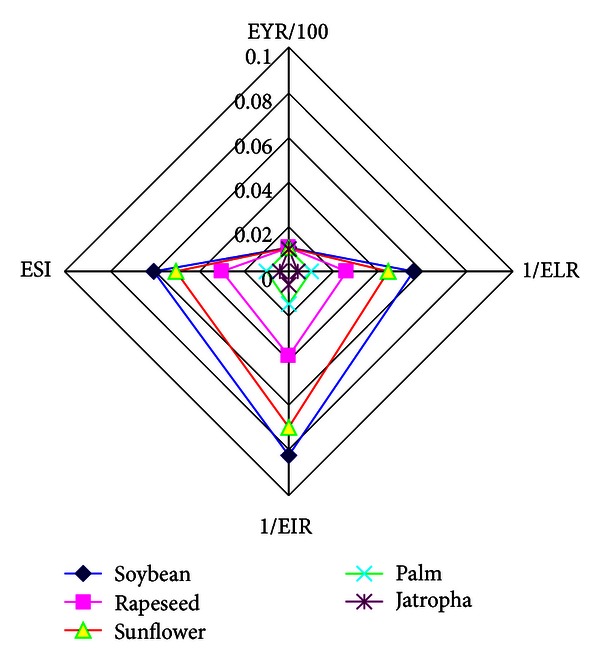
Multicriteria representations of various biodiesel production options.

 In order to analyze the emergy indices comprehensively, a multicriteria representation has been proposed, as shown in Figure 8. ESI and EYI are the-larger-the-better criteria, and the-smaller-the-better criteria such as EIR and ELR are transformed into the-larger-the-better criteria in reciprocal way. The sequence of the comprehensive performance of the systems from the best to the worst is soybean-, sunflower-, rapeseed-, palm-, and jatropha-based biodiesel option. This multi-criteria representation has neglected the comparison of transformities and the importance (weights) of the emergy indices; Section 3 proposes a novel methodology to analyze the integrated performance of the emergy indices comprehensively.

## 3. Sustainability Efficiency Analysis

### 3.1. Data Envelopment Analysis

**Figure 9:**
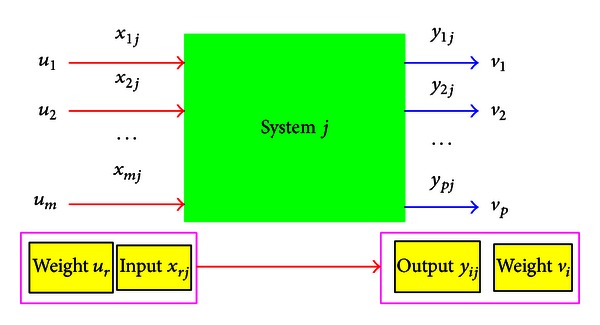
Structure of DEA assessment system.

The data envelopment was developed by Charnes et al. in 1978 [34], which was widely used for assessing the alternatives with the inputs and outputs of these systems [35]. Each alternative can be considered as a system which is also called decision-making unit (DMU), as shown in Figure 9.

The meaning of the symbols in Figure 9 has been defined as follows:

 
*r* : 1,2,…, *m* inputs,
 
*i*: 1,2,…, *p* outputs,
 
*j*: 1,2,…, *t* system *j*,
 
*x*_*rj*_: the amount of input *r* for unit *j*,
 
*y*_*ij*_: the amount of input *i* for unit *j*,
 
*u*_*r*_: the weighting of input *r*,
 
*v*_*i*_: the weighting of output *i*.

The efficiency of a decision-making unit *j* can be formulated by the ratio of weighted sum of outputs to weighted sum of inputs [36], as shown in (7):

(7) hj=∑i=1pviyij∑r=1murxrj

Weights of the inputs and outputs can be determined by the decision makers/stakeholders, but that is a subjective way; a model can achieve that in an objective way. The model is to maximize the efficiency of the target system with the constraints of the efficiencies of the other systems ≤1 [37]. The efficiency of the target system *j*_0_ can be calculated by solving the programming problem as follows:

(8) max⁡h0=∑i=1pviyij0∑r=1murxrj0

subject to

(9) ∑i=1pviyij∑r=1murxrj≤1 (j=1,2,…,t)ur≥ε r=1,2,…,mvi≥ε i=1,2,…,p,

where *ε* is a nonarchimedean construct.

The constraints (9) indicate that the upper bound of the efficiency of the DMU is 100%, namely, the efficiency cannot exceed 1.

The model can be transformed into matrix form:

(10) max⁡h0=vTy0uTx0,vTyjuTxj≤1,u≥ε, v≥ε,

where *u* = (*u*_1_, *u*_2_,…, *u*_*m*_)^*T*^, *v* = (*v*_1_, *v*_2_,…, *v*_*p*_)^*T*^, *x*_*j*_ = (*x*_1*j*_, *x*_2*j*_,…, *x*_*mj*_)^*T*^, *y*_*j*_ = (*y*_1*j*_, *y*_2*j*_,…, *y*_*pj*_)^*T*^,    *x*_0_ = (*x*_1*j*_0__, *x*_2*j*_0__,…, *x*_*mj*_0__)^*T*^,    *y*_0_ = (*y*_1*j*_0__, *y*_2*j*_0__,…, *y*_*pj*_0__)^*T*^.

Based on Charnes-Cooper transformation [38], the equivalent linear programming can be acquired:

(11) max⁡vTy0

subject to

(12) ∑r=1murxrj0=1,∑i=1pviyij−∑r=1murxrj≤0 (j=1,2,…,t),u≥ε, v≥ε

Then, the linear programming problem can be transformed into the following form:

(13) max⁡(uT,vT)(0y0),vTyj−uTxj≤0 (j=1,2,…,t),  u≥ε, v≥ε

According to the duality theory of linear programming, it can be transformed into the following form:

(14) min⁡g−ε(∑r=1msr++∑i=1psi−),

subject to

(15) ∑j=1txrjλj+sr−−gxrj0=0,∑j=1tyijλj−si+−yij0=0,λj≥0 (j=1,2,…,t),sr−≥0 (r=1,2,…,m),si+≥0 (i=1,2,…,p).

**Definition 1**

If the optimal value  *g* = 1, then the decision-making unit can be identified as weak DEA effective and vice versa.

**Definition 2**

If the optimal value  *g* = 1 and the solution satisfies *s*_*r*_^−^ = 0  (*r* = 1,2,…, *m*), *s*_*i*_^+^ = 0  (*i* = 1,2,…, *p*), then the decision-making unit can be identified as DEA effective, and vice versa.

Therefore, once the inputs and outputs of the systems for assessment have been obtained, the question whether some system is DEA efficient or not can be answered by solving the linear programming (14) and (15).

### 3.2. Sustainability Efficiency of Biodiesel Systems

The concept of sustainability has been defined as the ratio of the sum of the weighted outputs to the sum of the weighted inputs, and the inputs comprise transformity, environmental load ratio, and environmental investment ratio, the outputs comprise emergy index of sustainability, emergy yield ratio and product, as shown in (16):

(16) hj=v1ESI+v2EYR+v3Pu1Tr+u2ELR+u3EIR,

where *v*_1_, *v*_2_, and *v*_3_ represent the weights of ESI, EYR, and *P*, respectively; *u*_1_, *u*_2_, and *u*_3_ represent the weights of Tr, ELR and EIR respectively.

**Figure 10:**
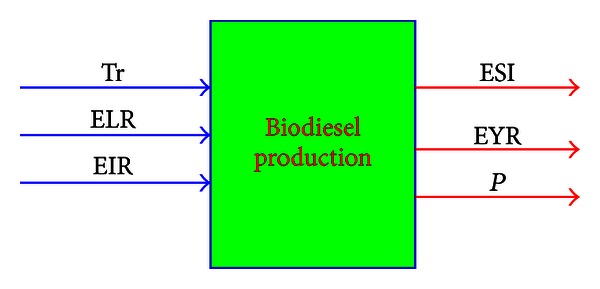
DEA assessment system for biodiesel production.

The production of biodiesel can be considered as a system; similarly, the alternatives for biodiesel production can also be considered as decision-making units (DMUs). The inputs of these DMUs comprise transformity (Tr), environmental loading ratio (ELR) and emergy investment ratio (EIR), the outputs include emergy sustainable index (ESI) emergy yield ratio (EYR) and product yield (*P*). The structure of DEA assessment system for biodiesel production is shown in Figure 10.

In order to calculate more conveniently, all the data including inputs and output should be processed in the following ways, as shown in (17):

(17) Xrj=xrj∑j=1txrj/t r=1,2,…,m;  j=1,2,…,t,

where *X*_*rj*_ is the (*j*) th input or output in the (*r*) th DMU; *t* is the total number of the DMUs.

Based on the data shown in Table 8 and the data processed method, the emergy indices involved in the DEA assessment model are shown in Table 9.

Based on the data shown in Table 9, the DEA assessment methodology can be utilized to measure the sustainability efficiency of each biodiesel production system; the calculating results including effective value, slack value and surplus value can be calculated, as shown in Table 10. According to Definitions 1 and 2, it can be summarized that the biodiesel production systems based on soybean, sunflower, and palm are DEA efficient, but the other two based on rapeseed or jatropha are non-DEA efficient.

Ye et al. had introduced a projection improvement analysis methodology to improve the non-DEA-efficient DMU to DEA efficient one [38]. Assume the optimal solution of linear programming (14) and (15) is *g* ^*j*^, *s*_*rj*_^−^, *s*_*ij*_^+^ for DMU  *j*  which is non-DEA-efficient, then the projection of the inputs and outputs on the relative efficient surface can be calculated using (18):

(18) x⌢rj=gjxrj−srj−y⌢ij=yij+sij+,

where ${\overset{⌢}{x}}_{rj}$ and ${\overset{⌢}{y}}_{ij}$ are the improved inputs and outputs, respectively.

From Table 10, it can be deduced that some of the inputs and outputs of the biodiesel production systems based on rapeseed and Jatropha should be improved to make them DEA efficient, the improvement results are calculated, as shown in Table 11, to rapeseed-based biodiesel production system, the emergy loading ratio should be reduced from 1.71 to 1.22, and simultaneously, it is necessary to increase the emergy index of sustainability and emergy yield ratio from 0.37 to 0.74 and from 0.83 to 0.93, respectively. Similarly, to jatropha-based biodiesel production system, some of the inputs, for instance, the transformity, emergy loading ratio and ecological footprint should be reduced from 1.70 to 1.05, from 0.59 to 0.54, and from 1.35 to 1.20, respectively, simultaneously, it is necessary to increase the emergy index of sustainability and emergy yield ratio from 1.41 to 1.64 and from 1.09 to 1.17, respectively. With these improvements, the rapeseed-based or jatropha-based biodiesel production system will be DEA efficient.

The sequence of the sustainability efficiency of the five kinds of crop-based biodiesel from the best to the worst is {soybean-based, sunflower-based, palm-based}, {jatropha-based}, and {rapeseed-based}.

## 4. Results and Discussion

Emergy analysis has the ability to integrate the environmental resources, purchased inputs, monetary, and labor into the generic indices of sustainability such as emergy index of sustainability and transformity.

Emergy analysis has been used to study the sustainability of soybean-, rapeseed-, sunflower-, jatropha- and palm-based biodiesel production options in this paper, the emergy indices of sustainability are 0.06, 0.03, 0.05, 0.01 and 0.004, respectively. It can be recognized that soybean-based biodiesel is the most sustainable and none of the five options can be recognized as sustainable in long terms.

The economic inputs occupy the most part in the total solar joules of each crop-based biodiesel system, and the dependence on purchased resources reduces the fraction of renewable resources and increases the environmental loads. Therefore, developing new technologies to reduce the use of purchased resources and increase the use of renewable is the best way to achieve sustainable development of biodiesel.

The transformities of soybean-based, rapeseed-based, sunflower-based, palm-based, and jatropha-based biodiesel productions are calculated to be 9.85*E* + 12 sej/kg, 9.18*E* + 12 sej/kg, 6.40*E* + 12 sej/kg, 5.83*E* + 12 sej/kg, and 1.61*E* + 13 sej/kg, respectively. In the current situation, palm-based biodiesel is the most emergy-saving option. In order to decrease the transformities, improving the plantation technology to reduce the use of fertilizers and pesticide and to increase the yield of crops is urgently needed.

In order to compare the emergy indices comprehensively, DEA has been used to analyze the sustainability efficiency, the inputs comprise transformity (Tr), environmental loading ratio (ELR), and emergy investment ratio (EIR), the outputs include emergy sustainable index (ESI) emergy yield ratio (EYR) and product yield (*P*). The biodiesel production system based on soybean, sunflower, and palm are DEA efficient, but the other two based on rapeseed or jatropha are non-DEA efficient.

Although soybean-, sunflower-, palm-based biodiesel options have been recognized as DEA efficient, due to the debate of “biodiesel versus food,” if soybean and sunflower are used to produce biodiesel, it may cause food crisis. From this point of view, palm is the most suitable to be chosen as the sustainability efficient option.

The authors calculate the emergy indices in an accurate and objective way, but there are also some drawbacks.

- The consistency of the data: the data about the energy consumption in each item and the transformity are cited from different published work, and some of the data has been adjusted.
- The temporal and spatial consistency: the data used in emergy indices calculation are not obtained in the same time, and the cases studied have been assumed to locate in the same region.

The future work is to obtain high quality data and develop related method to verify the accuracy of the data.

## 5. Conclusion

Although biodiesel has been a hot spot since several decades ago and different scales of biodiesel plants have been operated around the world, the sustainability is not optimistic in the current situations. None of the options for biodiesel production is sustainable in a long term. In order to achieve high sustainability of biodiesel production, new plantation technologies to reduce the use of fertilizers and pesticide and to increase the yield of crops and novel methods to produce biodiesel with vegetables oil are prerequisite.

## Figures and Tables

**Table 1:** Emergy indices and corresponding calculated methodology used in this paper.

Name (abbreviation)	Formula	Note
Renewable environmental resources	*R*	Renewable resources provided by the environment
Nonrenewable environmental resources	*N*	Nonrenewable resource
Economic inputs	*F*	The purchased emergy
Product (mass or energy)	*P*	The mass or energy of the product
Yield	*Y* = *R* + *N* + *F *	The output emergy
Transformity	Tr = *Y*/*P *	The ratio of the total emergy inputs to the mass or energy of the product
Emergy yield ratio	EYR = *Y*/*F *	The ratio of the output emergy to the purchased emergy
Environmental load ratio	ELR = (*F* + *N*)/*R *	The ratio of nonrenewable emergy plus the purchased emergy to the renewable environmental emergy
Environmental investment ratio	EIR = *F*/(*R* + *N*)	The ratio of the purchased emergy to the renewable environmental emergy plus the nonrenewable emergy
Emergy index of sustainability	ESI = EYR/ELR	The ratio of the emergy yield ratio to the environmental load ratio

**Table 2:** Calculation of the available energy of each item for soybean-based biodiesel.

Sunlight	Area = 23304.59 m^2^
	Insolation = 4.77*E* + 9 J/m^2^/yr
	Albedo = 0.3
	*T* = 0.35 yr
	Energy (J) = (Area) × (Insolation) × (1 − Albedo) × *T* = 1.17*E* + 13 J

Rain geopotential	Area = 23304.59 m^2^
	Rainfall = 0.3 m
	Rain-off rate = 0.20
	Average elevation = 100 m
	Density = 1000 kg/m^3^
	Gravity = 9.80 s/m^2^
	Energy (J) = (Area) × (Rainfall) × (Rainoff rate) × (Average elevation) × Density × Gravity = 1.37*E* + 09 J

Rain chemical potential	Area = 23304.59 m^2^
	Rainfall = 0.3 m
	Density = 1000 kg/m^3^
	Gibbs free energy = 1940 J/kg
	Energy (J) = (Area) × (Rainfall) × (Density) × (Gibbs free energy) = 1.36*E* + 10 J

Wind	Area = 23304.59 m^2^
	Air density = 1.23 kg/m^3^
	Drag coefficient = 0.001
	Average annual wind velocity = 2.4 m/s
	Geostrophic wind = 10 Average annual wind velocity/6 = 4.17 m/s
	*T* = 0.35 yr
	Energy (J) = (Area) × (Air Density) × (Drag coefficient) × (Geostrophic wind)^3^ × (3600 × 24 × 365 × *T*) = 2.29*E* + 10 J

Topsoil loss	Area = 23304.59 m^2^
	Topsoil loss energy = 1.32*E* + 09 J/hm^2^
	Energy (J) = (Area) × Topsoil loss energy = 3.08*E* + 09 J

**Table 3:** Emergy analysis table for soybean-based biodiesel.

Stage	Type	Item	Data	Reference	Transformity (sej/unit)	Reference	Solar emergy (sej)
Plantation and reap	*R*	Sunlight (J)	1.17*E* + 13	Calculated	1.00*E* + 00	[5]	1.17*E* + 13
		Rain geopotential (J)	1.37*E* + 09	Calculated	4.70*E* + 04	[5]	6.44*E* + 13
		Rain chemical potential (J)	1.36*E* + 10	Calculated	3.05*E* + 04	[5]	4.15*E* + 14
		Wind (J)	2.29*E* + 10	Calculated	1.50*E* + 03	[6]	3.44*E* + 13
	*N*	Topsoil loss	3.08*E* + 09	Calculated	7.40*E* + 04	[5]	2.28*E* + 14
	F	Water (kg)	5033790	Statistics	4.65*E* + 08	[6]	2.34*E* + 15
		Nitrogen (kg)	93.22	Statistics	2.40*E* + 13	[5]	2.24*E* + 15
		Biocide trifluralin (kg)	2.33	Statistics	1.48*E* + 13	[5]	3.45*E* + 13
		Pesticide pirimicarb (kg)	2.33	Statistics	1.48*E* + 13	[5]	3.45*E* + 13
		Pesticides dicofol (kg)	1.17	Statistics	1.48*E* + 13	[5]	1.73*E* + 13
		Diesel (kg)	212.5	Estimated	3.04*E* + 12	[7]	6.46*E* + 14
		Human labor (h)	200	Estimated	1.1*E* + 12	[5]	2.2*E* + 14
		Soybean seed ($)	108.56	Statistics	1.18*E* + 13	[8]	1.28*E* + 15

Soybean oil production	*F*	Crude oil (J)	1.05*E* + 10 J	Statistics	5.4*E* + 04	[9]	5.67*E* + 14
		Electricity (J)	1245.86*E* + 06	Statistics	3.36*E* + 05	[8]	4.19*E* + 14

Biodiesel production	*F*	MeOH (kg)	217.90	Statistics	1.76*E* + 12	[8]	3.84*E* + 14
		NaOH (kg)	8.16	Statistics	6.38*E* + 12	[8]	5.21*E* + 13
		H_2_O (kg)	1018.41	Statistics	4.65*E* + 08	[6]	4.74*E* + 11
		H_3_PO_4_ (kg)	6.06	Statistics	2.65*E* + 12	[9]	1.61*E* + 13
		Electricity (J)	251.69*E* + 06	Statistics	3.36*E* + 05	[8]	8.46*E* + 13
		Human labor (h)	800	Estimated	1.1*E* + 12	[5]	8.8*E* + 14

**Table 4:** Emergy analysis table for rapeseed-based biodiesel.

Stage	Type	Item	Data	Reference	Transformity (sej/unit)	Reference	Solar emergy (sej)
Plantation and reap	*R*	Sunlight (J)	0.52*E* + 13	Calculated	1.00*E* + 00	[5]	0.52*E* + 13
		Rain geopotential (J)	0.61*E* + 09	Calculated	4.70*E* + 04	[5]	2.87*E* + 13
		Rain chemical potential (J)	0.60*E* + 10	Calculated	3.05*E* + 04	[5]	1.83*E* + 14
		Wind (J)	1.01*E* + 10	Calculated	1.50*E* + 03	[6]	1.52*E* + 13
	*N*	Topsoil loss	1.36*E* + 09	Calculated	7.40*E* + 04	[5]	1.01*E* + 14
	*F*	Water (kg)	556010	Statistics	4.65*E* + 08	[6]	0.26*E* + 15
		Nitrogen (kg)	236.82	Statistics	2.40*E* + 13	[5]	5.69*E* + 15
		Biocide trifluralin (kg)	1.03	Statistics	1.48*E* + 13	[5]	1.53*E* + 13
		Pesticide pirimicarb (kg)	1.03	Statistics	1.48*E* + 13	[5]	1.53*E* + 13
		Diesel (kg)	127.5	Estimated	3.04*E* + 12	[5]	3.88*E* + 14
		Human labor (h)	200	Estimated	1.1*E* + 12	[5]	2.2*E* + 14
		Rapeseed seed ($)	24.32	Statistics	1.18*E* + 13	[8]	0.29*E* + 15

Rapeseed oil production	*F*	Crude oil (J)	0.45*E* + 10 J	Statistics	5.4	[9]	2.43*E* + 14
		Electricity (J)	939.66*E* + 06	Statistics	3.36*E* + 05	[8]	3.14*E* + 14

Biodiesel production	*F*	MeOH (kg)	216.23	Statistics	1.76*E* + 12	[8]	3.81*E* + 14
		NaOH (kg)	8.03	Statistics	6.38*E* + 12	[8]	5.13*E* + 13
		H_2_O (kg)	1019.36	Statistics	4.65*E* + 08	[6]	4.74*E* + 11
		H_3_PO_4_ (kg)	6.28	Statistics	2.65*E* + 12	[9]	1.81*E* + 13
		Electricity (J)	2.36*E* + 08	Statistics	3.36*E* + 05	[8]	7.94*E* + 13
		Human labor (h)	800	Estimated	1.1*E* + 12	[5]	8.8*E* + 14

**Table 5:** Emergy analysis table for sunflower-based biodiesel.

Stage	Type	Item	Data	Reference	Transformity (sej/unit)	Reference	Solar emergy (sej)
Plantation and reap	*R*	Sunlight (J)	0.60*E* + 13	Calculated	1.00*E* + 00	[5]	0.60*E* + 13
		Rain geopotential (J)	0.70*E* + 09	Calculated	4.70*E* + 04	[5]	3.29*E* + 13
		Rain chemical potential (J)	0.70*E* + 10	Calculated	3.05*E* + 04	[5]	2.14*E* + 14
		Wind (J)	1.18*E* + 10	Calculated	1.50*E* + 03	[6]	1.77*E* + 13
	*N*	Topsoil loss	1.95*E* + 09	Calculated	7.40*E* + 04	[5]	1.44*E* + 14
	*F*	Water (kg)	1.30*E* + 06	Statistics	4.65*E* + 08	[6]	0.61*E* + 15
		Nitrogen (kg)	83.89	Statistics	2.40*E* + 13	[5]	2.02*E* + 15
		Biocide trifluralin (kg)	1.20	Statistics	1.48*E* + 13	[5]	1.78*E* + 13
		Diesel (kg)	326.3	Estimated	3.04*E* + 12	[5]	9.93*E* + 14
		Human labor (h)	200	Estimated	1.1*E* + 12	[5]	2.20*E* + 14
		Sunflower seed ($)	120.30	Statistics	1.18*E* + 13	[8]	1.43*E* + 15

Sunflower oil production	*F*	Crude oil (J)	0.71*E* + 10	Statistics	5.4*E* + 04	[10]	3.83*E* + 14
		Electricity (J)	9.17*E* + 08	Statistics	3.36*E* + 05	[8]	3.08*E* + 14

Biodiesel production	*F*	MeOH (kg)	217.76	Statistics	1.76*E* + 12	[8]	3.84*E* + 14
		NaOH (kg)	8.03	Statistics	6.38*E* + 12	[8]	5.13*E* + 13
		H_2_O (kg)	1018.34	Statistics	4.65*E* + 08	[6]	4.74*E* + 11
		H_3_PO_4_ (kg)	6.23	Statistics	2.65*E* + 12	[9]	1.80*E* + 13
		Electricity (J)	215.72*E* + 06	Statistics	3.36*E* + 05	[8]	7.25*E* + 13
		Human labor (h)	800	Estimated	1.1*E* + 12	[5]	8.8*E* + 14

**Table 6:** Emergy analysis table for palm-based biodiesel.

Stage	Type	Item	Data	Reference	Transformity (sej/unit)	Reference	Solar emergy (sej)
Plantation and reap	*R*	Sunlight (J)	0.13*E* + 13	Calculated	1.00*E* + 00	[5]	0.13*E* + 13
		Rain geopotential (J)	0.15*E* + 09	Calculated	4.70*E* + 04	[5]	0.71*E* + 13
		Rain chemical potential (J)	0.15*E* + 10	Calculated	3.05*E* + 04	[5]	0.46*E* + 14
		Wind (J)	0.25*E* + 10	Calculated	1.50*E* + 03	[6]	0.38*E* + 13
	*N*	Topsoil loss	0.33*E* + 09	Calculated	7.40*E* + 04	[5]	2.44*E* + 13
	*F*	Nitrogen (kg)	39.03	Statistics	2.40*E* + 13	[5]	9.37*E* + 14
		Phosphate (kg)	8.71	Statistics	2.02*E* + 13	[5]	1.76*E* + 14
		Potash (kg)	150.32	Statistics	1.74*E* + 12	[5]	2.62*E* + 14
		Biocide Paraquat (kg)	0.26	Statistics	1.48*E* + 13	[5]	0.32*E* + 13
		Diesel (J)	6.44*E* + 08	Statistics	1.11*E* + 05	[5]	7.15*E* + 13
		Human labor (h)	200	Estimated	1.1*E* + 12	[5]	2.2*E* + 14
		Palm management fee ($)	225.56	Estimated	1.18*E* + 13	[8]	2.66*E* + 15

Palm oil production	*F*	Crude oil (J)	9.13*E* + 08	Estimated	5.4*E* + 04	[10]	4.93*E* + 13
		Electricity (J)	2.69*E* + 08	Statistics	3.36*E* + 05	[8]	9.06*E* + 13

Biodiesel production	*F*	MeOH (kg)	180	Statistics	1.76*E* + 12	[8]	3.17*E* + 14
		NaOH (kg)	5.86	Statistics	6.38*E* + 12	[8]	3.74*E* + 13
		H_2_O (kg)	1500	Estimated	4.65*E* + 08	[6]	6.98*E* + 11
		H_3_PO_4_ (kg)	0.96	Estimated	2.65*E* + 12	[9]	0.28*E* + 13
		Electricity (J)	1.14*E* + 08	Statistics	3.36*E* + 05	[8]	3.82*E* + 13
		Human labor (h)	800	Estimated	1.1*E* + 12	[5]	8.8*E* + 14

**Table 7:** Emergy analysis table for jatropha-based biodiesel.

Stage	Type	Item	Data	Reference	Transformity (sej/unit)	Reference	Solar emergy (sej)
Plantation and reap	*R*	Sunlight (J)	0.15*E* + 13	Calculated	1.00*E* + 00	[5]	0.15*E* + 13
		Rain geopotential (J)	0.17*E* + 09	Calculated	4.70*E* + 04	[5]	0.80*E* + 13
		Rain chemical potential (J)	0.17*E* + 10	Calculated	3.05*E* + 04	[5]	0.52*E* + 14
		Wind (J)	0.28*E* + 10	Calculated	1.50*E* + 03	[6]	0.42*E* + 13
	*N*	Topsoil loss	0.38*E* + 09	Calculated	7.40*E* + 04	[5]	2.81*E* + 13
	F	Water (kg)	10705350	Statistics	4.65*E* + 08	[6]	4.98*E* + 15
		Nitrogen (kg)	87	Statistics	2.40*E* + 13	[5]	2.09*E* + 15
		Phosphate (kg)	174	Statistics	2.02*E* + 13	[5]	3.51*E* + 15
		Diesel (kg)	255	Estimated	3.04*E* + 12	[5]	7.75*E* + 14
		Human labor (h)	200	Estimated	1.1*E* + 12	[5]	2.2*E* + 14
		Jatropha management fee ($)	225.56	Estimated	1.18*E* + 13	[8]	2.66*E* + 15

Jatropha oil production	*F*	Electricity (J)	4.91*E* + 08	Statistics	3.36*E* + 05	[8]	1.65*E* + 14

Biodiesel production	*F*	MeOH (kg)	391	Statistics	1.76*E* + 12	[8]	6.88*E* + 14
		NaOH (kg)	8	Statistics	6.38*E* + 12	[8]	5.10*E* + 13
		H_2_O (kg)	1000	Estimated	4.65*E* + 08	[6]	4.65*E* + 11
		H_3_PO_4_ (kg)	6.84	Statistics	2.65*E* + 12	[9]	1.81*E* + 13
		Electricity (J)	3.02*E* + 07	Statistics	3.36*E* + 05	[8]	1.01*E* + 13
		Human labor (h)	800	Estimated	1.1*E* + 12	[5]	8.8*E* + 14

**Table 8:** Emergy indices of various crops-based biodiesel.

Emergy indices	Tr (sej/kg)	EYR	ELR	EIR	ESI
Soybean	9.95*E* + 12	1.08	17.92	12.20	0.06
Rapeseed	9.18*E* + 12	1.04	38.58	26.58	0.03
Sunflower	6.40*E* + 12	1.07	22.60	14.41	0.05
Palm	5.83*E* + 12	1.01	99.55	69.61	0.01
Jatropha	1.61*E* + 13	1.006	244.68	171.08	0.004

**Table 9:** The processed data of the inputs and outputs of DEA assessment system.

Crop	Inputs			Outputs		
	Tr	ELR	EIR	ESI	EYR	P
Soybean	1.05	0.54	0.53	1.64	1.17	1.00
Rapeseed	0.97	1.71	1.71	0.37	0.83	1.00
Sunflower	0.67	0.71	0.69	1.13	1.06	1.00
Palm	0.61	1.45	1.52	0.44	0.85	1.00
Jatropha	1.70	0.59	0.56	1.41	1.09	1.00

**Table 10:** The calculating results: effective value, slack value, and surplus value.

	Effective value	Slack or Surplus value					
	*g*	*s*_1_^−^	*s*_2_^−^	*s*_3_^−^	*s*_1_^+^	*s*_2_^+^	*s*_3_^+^
Soybean	1	0	0	0	0	0	0
Rapeseed	0.6583	0	0.0272	0	0.3979	0.1198	0
Sunflower	1	0	0	0	0	0	0
Palm	1	0	0	0	0	0	0
Jatropha	0.9464	0.5589	0.0184	0	0.2300	0.0800	0

**Table 11:** Improved objective value of Non-DEA effective crop-based biodiesel system.

	Tr		ELR		ESI		EYR	
	Actual	Improved	Actual	Improved	Actual	Improved	Actual	Improved
Rapeseed	—	—	1.71	1.0985	0.37	0.7679	0.83	0.9498
Jatropha	1.70	1.0500	0.59	0.5400	1.41	1.64	1.09	1.17
